# Distribution and Characterization of Deep Rhodolith Beds off the Campania coast (SW Italy, Mediterranean Sea)

**DOI:** 10.3390/plants9080985

**Published:** 2020-08-04

**Authors:** Francesco Rendina, Sara Kaleb, Annalisa Caragnano, Federica Ferrigno, Luca Appolloni, Luigia Donnarumma, Giovanni Fulvio Russo, Roberto Sandulli, Valentina Roviello, Annalisa Falace

**Affiliations:** 1 Department of Science and Technology, University of Naples “Parthenope”, URL CoNISMa, Centro Direzionale, Is. C4, 80143 Naples, Italy; federica.ferrigno@uniparthenope.it (F.F.); luca.appolloni@uniparthenope.it (L.A.); luigia.donnarumma@uniparthenope.it (L.D.); giovanni.russo@uniparthenope.it (G.F.R.); roberto.sandulli@uniparthenope.it (R.S.); 2 Department of Life Sciences, University of Trieste, Via L. Giorgieri 10, I-34127 Trieste, Italy; skaleb@units.it (S.K.); annalisacaragnano@hotmail.com (A.C.); falace@units.it (A.F.); 3 Department of Chemical, Materials and Production Engineering, University of Naples “Federico II”, Piazzale Tecchio 80, 80125 Naples, Italy; valentina.roviello@gmail.com

**Keywords:** rhodolith beds, coralline algae, maerl, ROV, biogenic habitats, Tyrrhenian Sea

## Abstract

Rhodolith beds (RBs) are bioconstructions characterized by coralline algae, which provide habitat for several associated species. Mediterranean RBs are usually located in the mesophotic zone (below 40 m), and thus are frequently remote and unexplored. Recently, the importance and vulnerability of these habitats have been recognized by the European Community and more attention has been drawn to their investigation and conservation. This study reports the results of an extensive monitoring program, carried out within the Marine Strategy Framework Directive (2008/56/EC), in six sites off the Campania coast (Italy, Mediterranean Sea). New insights were given into the distribution, cover, vitality (i.e., live/dead rhodolith ratio), structural complexity, and coralline algae composition of RBs. Remotely operated vehicles (ROV) investigations allowed the description of several RBs, and the discovery of a RB with rhodolith cover >65% offshore the Capri Island. Only two sites (Secchitiello and Punta Campanella) showed a very low mean cover of live rhodoliths (<10%); hence, not being classifiable as RBs. The collected rhodoliths were mostly small pralines (~2 cm), spheroidal to ellipsoidal, with growth-forms ranging from encrusting/warty to fruticose/lumpy. Coralline algae identification revealed a high diversity within each bed, with a total of 13 identified taxa. The genus *Lithothamnion* dominated all sites, and *Phymatolithon calcareum* and *Lithothamnion corallioides*, protected by the Habitats Directive (92/43/EEC), were detected in all RBs.

## 1. Introduction

Rhodolith beds (RBs) are biogenic habitats formed by unattached, non-geniculate coralline algae (CA; Corallinophycidae, Rhodophyta) [1]. Rhodolith sizes range from 1 to 10 cm, varying from highly branched to roundish shapes [2,3,4]. These habitats have a worldwide distribution, having been described in tropical [5,6,7], temperate [8,9,10,11], and polar [12,13,14] regions, from the low intertidal zone to depths over 150 m [4,15].

RBs are ecosystem engineers, which create 3D biogenic habitats [1] for epiphytes and benthic invertebrates (both epifauna and infauna) [16,17,18], supporting higher species richness than nearby sedimentary habitats [3,4,19,20]. They provide critical ecosystem services, being settlement sites and nursery grounds for species of commercial interest [4,21]. In some rare cases, RBs are exploited through direct extraction for soil improvement [22] or may undergo indirect extraction as relict sand for beach nourishment by dredging [23]. RBs are also relevant in climate regulation, through their role in CO_2_ uptake, primary production, and carbonate production [1,24]. Moreover, through their CaCO_3_ production and dissolution, they contribute to the carbonate cycle of continental shelf ecosystems [6,25].

RBs are threatened by habitat degradation and loss of structural heterogeneity due to anthropogenic impacts, which can hamper the functioning of these habitats [26]. Rhodoliths are often fragile (particularly unattached branches), and their growth rate is on the order of 1 mm year^−1^, also depending on environmental factors as light and temperature [4,24,27]. For these reasons, RBs are highly affected by mechanical damage due to extraction of calcareous sediments and bottom fisheries [28,29,30,31]. Fish farms and aquaculture can threat these habitats too, by enhancing sedimentation rates and water turbidity [32,33]. Finally, CA, and consequentially RBs, are likely to be negatively affected by both ocean warming and acidification [24,34,35,36].

RBs have been classified as a non-renewable resource [1,37,38,39], and their vulnerability has been recognized worldwide though the adoption of several protection instruments. The Habitats Directive (92/43/EEC) [40] included the two species, *Phymatolithon calcareum* (Pallas) W.H.Adey and D.L.McKibbin ex Woelkering and L.M.Irvine and *Lithothamnion corallioides* (P.Crouan and H.Crouan) P.Crouan and H.Crouan, among those species subjected to exploitation and for which Member States have to ensure effective conservation measures. Mediterranean RBs have been recently included among the habitats of special interest within the Marine Strategy Framework Directive (MSFD,-2008/56/EC) [41,42], aiming at achieving the “Good Environmental Status” (GES) of all marine waters by 2020. Thus, the assessment of RBs distribution and biodiversity has been included within the protocol to evaluate the GES [1].

In the Mediterranean Sea, RBs, together with *Posidonia* meadows, coralligenous and vermetid bioconstructions, are considered as marine benthic habitats of high conservation interest and hotspots of biodiversity [43,44,45,46,47,48,49,50,51,52,53,54,55]. They are typically found around islands and capes, on submarine plateaux, seamounts, marine terraces, channels and banks [9,53], and frequently occur in the mesophotic zone, mostly at about 30–75 m depth [4], with the deepest bed discovered on the top of a seamount in the Balearic Sea (~150 m) [15]. They rarely occur at shallower depths, as for Atlantic RBs [1,53].

The current knowledge of their geographic distribution is still fragmented and incomplete, resulting in a wide but patchy distribution along the Mediterranean coasts [53]. This lack of information is due to the remoteness of these habitats, and consequently to expensive and time-consuming sampling efforts [1,56]. Remotely operated vehicles (ROVs) are fundamental instruments currently employed to explore and monitor deep habitats, with non-destructive and standardized protocols [57,58,59,60,61], or to evaluate the impact of fishing activities in coastal ecosystems [62,63,64]. ROV video/image analyses have been included in the monitoring protocol of RBs within the MSFD, in order to obtain useful data on the RB distribution and status [1]. Nevertheless, this method does not provide reliable information on RB coralline species compositions [1].

Mediterranean RBs are characterized by a higher CA biodiversity than NE Atlantic beds, which are usually monospecific/oligospecific, and mainly composed by *Phymatolithon calcareum* and *Lithothamnion corallioides* [53,65]. Moreover, RBs can be structured by a suite of combinations of rhodolith shapes and growth-forms [1,53], depending on the species, sedimentation, hydrodynamism, and seabed morphology [8,66,67], concurring to increase the RB complexity, which in turn influences the diversity and abundance of associated assemblages [3,19,68].

This study reports the results of an extensive regional-scale monitoring program, carried out within the MSDF, to provide new insights into the distribution, ecological status, and CA composition of deep Mediterranean RBs occurring off the Campania coast (Italy).

## 2. Materials and Methods

### 2.1. Study Area

**Figure 1:**
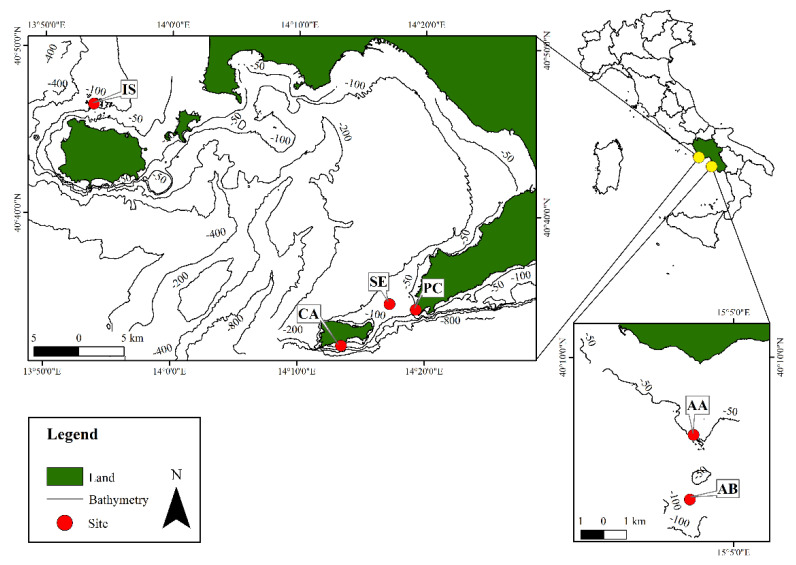
Map of the investigated sites along the Campania coast (Tyrrhenian Sea, Italy). Gulf of Naples’ sites: Capri (CA), Punta Campanella (PC), Secchitiello (SE), and Ischia (IS). Cilento coast’ sites: Acciaroli A (AA) and B (AB).

Surveys were carried out in 2017 and 2018, at depths ranging from 42 to 78 m, in six sites off the Campania coast (SW Italy, Mediterranean Sea), within the Italian monitoring program of the MSFD. Sites were chosen based both on previous records of coralline algal deposits along the Campania coast [69,70,71,72], and on the empirical knowledge of fishermen. Moreover, morpho-bathymetric data (multibeam echosounder and side-scan sonar) were used to corroborate the sites selection. Four sites were selected within the Gulf of Naples: Capri, Punta Campanella, Secchitiello, and Ischia; and two sites off the Cilento coast: Acciaroli A and Acciaroli B (Figure 1, Table 1).

### 2.2. Remote Data Acquisition and Analysis

**Figure 2:**
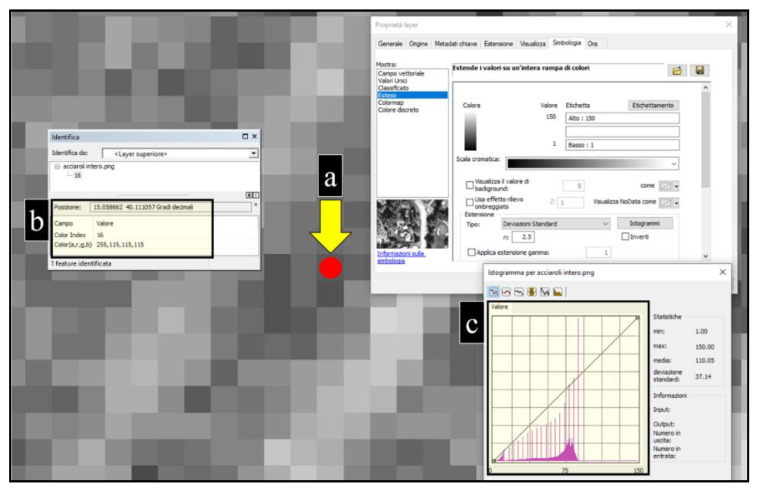
Example of rhodolith deposits definition by ArcGis 10.7.1 software (Acciaroli B site): (**a**) ROV point on backscatter pixel. (**b**) Colour index and associated RGBA. (**c**) Frequency histograms of pixels’ colour index in the side-scan sonar (SSS) image (frequencies range from 0 to 90).

Seafloor data were collected both by a Reson Sea Bat 8125 Multi-Beam Echo-Sounder (MBES) and a Side-Scan Sonar (SSS) Klein 3900 dual frequency system (100/500 kHz), which provided imagery with a high level of seabed resolution (100% coverage possible). SSS images were used to evaluate a possible extent of the rhodolith deposits on the base of sediment texture [73,74]. In fact, SSS backscatter is shadowed according to a grey scale of pixels. Each shade corresponds to a combination of RGBA colour (i.e., black: 0,0,0,255; white: 255,255,255,255), which is in turn associated to a colour index, varying from 0 (black, highest backscatter signal) to 150 (white, lowest backscatter signal). Comparing SSS with ROV images and direct samples of seabed, rhodolith deposits resulted to be associated to colour indices ranging between 0 and 90 (Figure 2). Pixels within this colour index range were automatically selected. The analysis was carried out using ArcGis 10.7.1 software.

Seabed videos were made through a ROV (“Perseus” of Ageotec) equipped with a high definition video camera (DVS-3000 high definition), two lights, two parallel laser beams at the fixed distance of 14.5 cm for the evaluation of the image surface. The ROV also hosted a navigation camera with underwater positioning system USBL (Ultra Short Base Line System), interfaced with the on-board navigation system, which allows to determine the real time geographical position and the ROV depth. Moreover, in order to estimate potential changes in time within the same area, reconstruction of the ROV routes was performed with the aim of being replicated in future monitoring programs.

At each site, three ROV video-routes were carried out, with a length of ca. 200 m each and a distance of at least 50 m from each other, covering a surface of ca. 1800 m^2^, calculated from the ROV routes’ length and the ROV camera field of vision (ca. 0.5 m). ROV videos were displayed using the software VisualSoft^®^, allowing the vision of HD videos with overlay of navigation data. A visual assessment of the sea bottom, characterized by rhodolith occurrence, was carried out on 60 images for site, for a total of 360 video frames analysed. The video frames were obtained extrapolating images (each 10 s) from the video tracks using the software DVDVideoSoft^®^. Videos’ and photos’ analyses were carried out according to the monitoring protocol for deep Mediterranean RBs, developed within the MSFD [75]. In particular, the total percentage cover (both live and dead thalli), the vitality (i.e., live vs. dead ratio [76]), and the predominant morphotypes (i.e., pralines, boxwork rhodoliths, and unattached branches [10]) of rhodoliths were analysed. The total (live and dead thalli) percentage cover is a more complete data with respect to the only live thalli cover (considered by MSFD protocol [75]), which does not allow to evaluate the eventual presence of RBs characterized by only dead thalli. Of course, the live thalli cover can be easily calculated by our data, since the live/dead rhodolith ratio was also reported.

In order to carry out bathymetric analyses, video frames from each site were divided into two depth intervals: “shallow”, within 40 and 59 m, and “deep”, within 60 and 79 m, while to test whether there was a significant effect of bottom sediment type on rhodolith cover and vitality, video frames were divided into two main categories: “fine” and “coarse” sediments (Table 2). The influence of depth and sediment on rhodolith cover and live/dead rhodolith ratio was assessed using univariate techniques. Data, checked with the Shapiro–Wilk’s test, were not normally distributed; thus, non-parametric tests were applied. Specifically, univariate PERMANOVAs based on similarity matrixes computed using Euclidean distances [77,78] were applied to avoid any assumption about the distribution of the variables [79]. Pairwise tests were run to estimate differences between pairs of sites. Analyses were performed using PAST software for Windows, version 3.16 [80]. Results are expressed as mean ± standard deviation (SD), and *p* is the significance.

### 2.3. Collection and Morphological Characterization of Rhodoliths

Three random samples were collected using a 25 l Van-Veen grab within each site, in the spots detected via ROV characterized by the highest rhodolith cover. Collected rhodoliths were cleaned to remove sediment and epiphytic organisms, air dried for at least 48 h and stored in the dark in zipper bags with silica gel.

Rhodoliths were classified following MSFD protocol [75] in pralines, boxwork rhodoliths, and unattached branches morphotypes. Results on rhodolith morphology were then plotted in a ternary diagram using the software Origin.

Rhodolith shapes were obtained by measuring the long (L), intermediate (I), and short (S) axes [81]. This provided data for plotting shapes on Sneed and Folk’s pebble shape diagram [82]. Finally, the rhodolith size was calculated using the volume of an ellipsoid [8], and data were reported in box plots using Origin software.

### 2.4. Coralline Algal Identification

A preliminary sorting of the CA samples was carried out at the stereomicroscope. Taxonomic identifications were carried out by scanning electron microscope SEM (Nova NanoSem 450-FEI-Thermo Fisher, Scientific, Waltham, MA, USA), following [83]. The identification of non-geniculate red algae follows the specialized literature and algal taxonomy follows Algaebase ([84] and references therein).

After the sorting, each sample was disposed on a 35 × 35 cm^2^ square, and then photographed to obtain high resolution images. For each square/image, the percentage cover of each taxon was estimated by the image analysis software VidAna 1.0 [85].

## 3. Results

### 3.1. Remote Data

**Figure 3:**
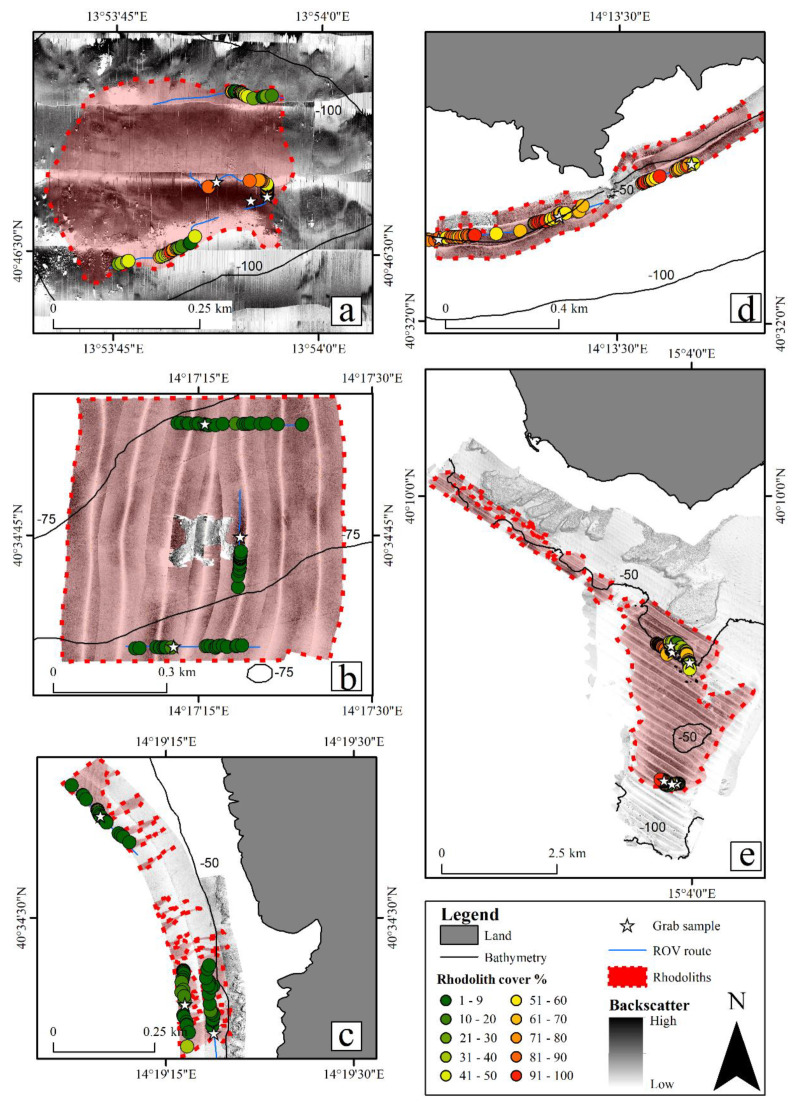
Side-scan sonar images overlaid with maps for the sites of Ischia (**a**), Secchitiello (**b**), Punta Campanella (**c**), Capri (**d**), and Acciaroli A and B (**e**), showing a possible extension of the rhodolith deposits. Red polygons localize the rhodolith deposits. Dotted lines mean that the rhodolith deposits could be wider with respect to the established boundaries. Blue lines and white stars indicate ROV routes and grab sampling points, respectively. Coloured dots indicate the total (live and dead) rhodolith cover (%) of each ROV video frame.

Rhodolith deposits were found at all the investigated sites, although their cover varied among sites. SSS data, together with ROV data and direct seabed samples, allowed the evaluation of the areal extension of each rhodolith deposit (Figure 3). The wider investigated deposit was the one off the Cilento coast, covering an area of ca. 8.49 km^2^, where the two sites Acciaroli A and B are present (Figure 3e). It was followed by Secchitiello (ca. 0.37 km^2^; Figure 3b), Capri (ca. 0.13 km^2^; Figure 3d), Ischia (ca. 0.11 km^2^; Figure 3a), and Punta Campanella (ca. 0.03 km^2^; Figure 3c).

**Figure 4:**
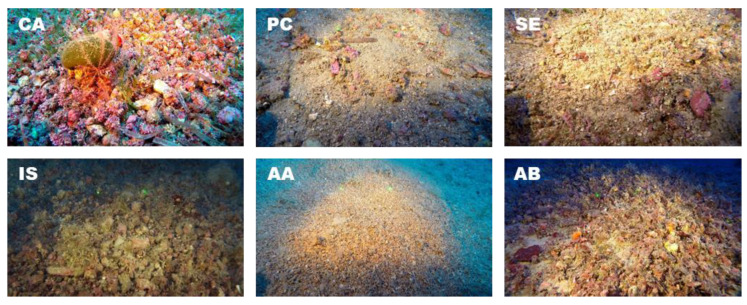
Representative video frames of the explored sites. In each video frame, the 2 green parallel laser beams indicate a fixed distance of 14.5 cm. CA, Capri; PC, Punta Campanella; SE, Secchitiello; IS, Ischia; AA, Acciaroli A; AB, Acciaroli B.

**Figure 5:**
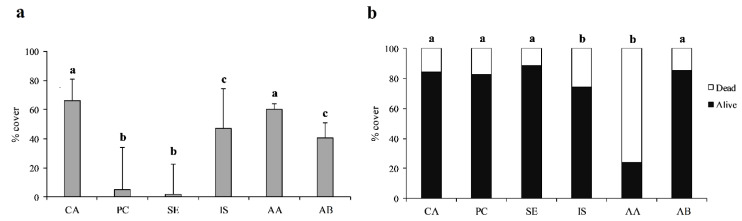
(**a**) Percentage of total (live and dead) rhodolith cover (± SD) at the investigated sites. (**b**) Percentage of live vs. dead thalli at each site. CA, Capri; PC, Punta Campanella; SE, Secchitiello; IS, Ischia; AA, Acciaroli A; AB, Acciaroli B. Different letters (a, b, and c) indicate significant differences between sites.

Depth significantly affected rhodolith cover (PERMANOVA, *p* < 0.001), with higher cover values detected in shallower waters (40–59 m). Likewise, cover was significantly influenced by sediment typologies (PERMANOVA, *p* < 0.001), with higher values in concurrence with coarse sediment. Significant differences in terms of rhodolith cover were also found among sites (PERMANOVA, *p* < 0.001). Video frames showed that the two shallower sites (i.e., Capri and Acciaroli A; Table 1) were characterized by the highest mean rhodolith cover (66 ±14 and 60 ±4%, respectively; Figure 4, Figure 5a). Punta Campanella and Secchitiello showed mean rhodolith cover lower than 10% (5 ± 28 and 2 ± 21%, respectively). Intermediate cover values were registered at Ischia and Acciaroli B (47 ± 27 and 40 ± 10%, respectively).

No significant difference in live/dead rhodolith ratio was detected between the two depth intervals and the two substrate types considered (PERMANOVA, *p* = 0.163 and 0.156, respectively). Significant differences in terms of live/dead rhodolith ratio were found among the explored sites (PERMANOVA, *p* < 0.001). In particular, the lowest percentages of dead thalli were observed at Capri (16 ± 8%), Punta Campanella (17 ± 12%), Secchitiello (12 ± 17%), and Acciaroli B (14 ± 9%) sites; the highest percentages of dead thalli were detected at Ischia and Acciaroli A sites (26 ± 15 and 76 ± 8%, respectively; Figure 5b).

ROV data showed that pralines were the prevalent morphotype characterizing Capri, Punta Campanella, Secchitiello, and Acciaroli B. At Ischia, the prevalent morphotype was pralines in 73% of the analysed video frames, while boxwork rhodoliths were the most abundant morphotype in the remaining 27% of frames. Finally, Acciaroli A was characterized by a prevalence of unattached branches in all the video frames.

### 3.2. Rhodolith Morphology

**Figure 6:**
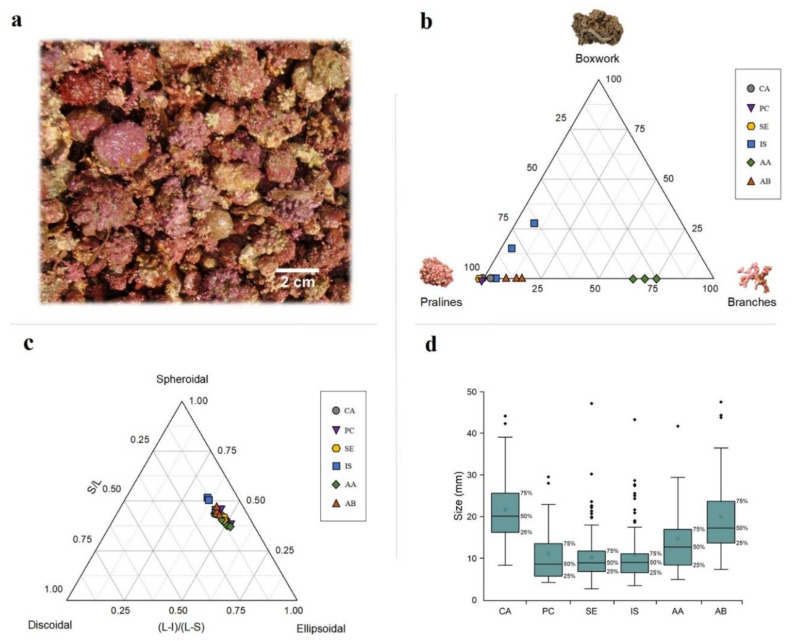
Collected rhodoliths and graphic analyses of their morphology, shape, and size. (**a**) Detail of collected rhodoliths from MS18-CA1 sampling, from Capri Island, 59 m depth. (**b**) Ternary plot of the main morphotypes (i.e., pralines, boxwork rhodoliths, and unattached branches). Each symbol indicates one of the three random samplings collected within each site. (**c**) Shape classification of rhodoliths using Sneed and Folk’s pebble shape diagram [82]. Long (L), intermediate (I), and short (S) axes of rhodoliths. (**d**) Box plot showing size range of rhodoliths calculated as the volume of an ellipsoid, following [8]; hence, unattached branches were not considered for size measurements. *n* = 120, except Acciaroli A, where *n* = 45. CA, Capri; PC, Punta Campanella; SE, Secchitiello; IS, Ischia; AA, Acciaroli A; AB, Acciaroli B.

Rhodolith growth forms varied from fruticose/lumpy to warty and encrusting (Figure 6a). Pralines were the predominant morphotype within all sites (except Acciaroli A) (Figure 6b), confirming the observations acquired by the ROV. In detail, this morphotype was the only found in all the samples collected at Punta Campanella and Secchitiello. Capri was mostly characterized by pralines too, even if few unattached branches (<5%) were also collected in one of the three samples. Ischia was the only site where multi-specific boxwork rhodoliths were collected, although the prevalent morphotype was pralines (>70% in all samples), followed by boxwork rhodoliths and unattached branches. Finally, Acciaroli A was characterized by the dominance of fragmented (0.6–10 mm length and 0.2–0.3 mm diameter) and brownish unattached branches (>70% in all samples), followed by pralines; Acciaroli B was primarily composed by pralines (>84% in all samples), and then by unattached branches.

The sphericity diagram showed a majority of ellipsoidal to spheroidal shapes for all sites (Figure 6c). The mean rhodolith size was relatively small in all sites, ranging from 21.8 mm ±8.5 in Capri to 10.6 mm ±6.1 in Secchitiello (Figure 6d).

### 3.3. Taxonomic Composition of Coralline Algae

**Figure 7:**
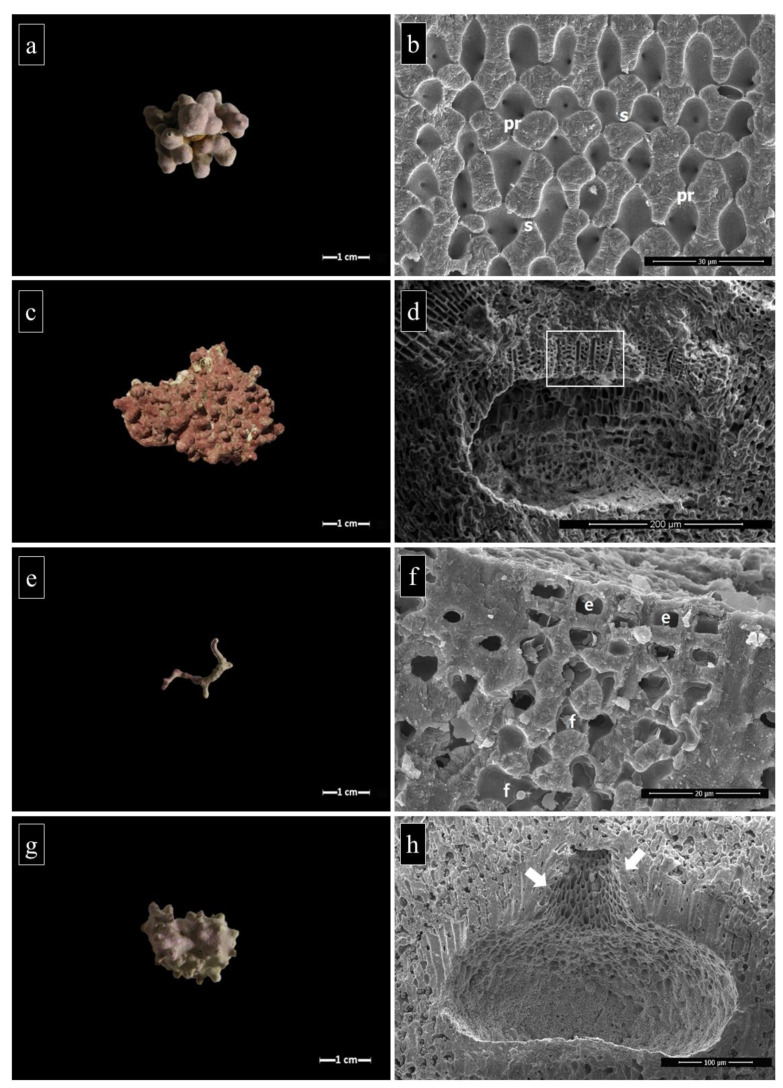
Examples of rhodolith specimens’ morphology and SEM images of their inner structure. (**a**) Fruticose rhodolith of *Lithophyllum racemus* from Ischia (specimen IS-12). (**b**) Perithallus of *L. racemus* (AB-12). Note the primary (pr) and secondary (s) pit connections. (**c**) Warty rhodolith of *Lithothamnion minervae* from Capri (specimen CA-33). (**d**) Multiporate conceptacle of *L. minervae* (CA-33). Note the pore canals (rectangle). (**e**) Unattached branch of *Phymatolithon calcareum* from Acciaroli B (specimen AB-03). (**f**) Epithallus and perithallus of *P. calcareum* (AB-03), with dome-shaped epithallial cells (e) and cell fusions (f). (**g**) Warty rhodolith of *Spongites fruticulosus* from Acciaroli B (specimen AB-07). **h.** Uniporate sporangial conceptacle of *S. fruticulosus* (AB-07). Note the cells protruding laterally into the pore canal (arrows).

Most of the rhodoliths observed during the sorting were monospecific, although some oligospecific samples occurred (i.e., boxwork rhodoliths). Thirteen CA taxa were identified, 10 of which at the species level: *Lithophyllum racemus* (Lamarck) Foslie (Figure 7a,b), *Lithophyllum* sp., *Lithothamnion corallioides* (P.Crouan and H.Crouan) P.Crouan and H.Crouan, *Lithothamnion crispatum* Hauck, *Lithothamnion minervae* Basso (Figure 7c,d), *Lithothamnion sonderi* Hauck, *Lithothamnion valens* Foslie, *Lithothamnion* spp., *Mesophyllum* sp., *Neogoniolithon hauckii* (Rothpletz) R.A.Townsend Huisman (ex *Neogoniolithon mamillosum)*, *Phymatolithon calcareum* (Pallas) W.H.Adey and D.L.McKibbin ex Woelkering and L.M.Irvine (Figure 7e,f), *Spongites fruticulosus* Kützing (Figure 7g,h), and *Titanoderma pustulatum* (J.V.Lamouroux) Nägeli.

**Figure 8:**
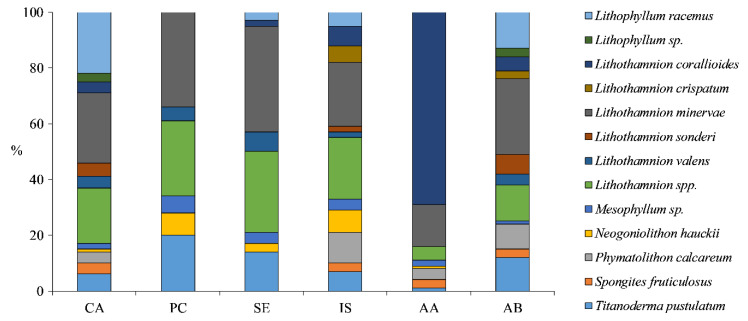
Coverage (%) of CA taxa at each site. CA, Capri; PC, Punta Campanella; SE, Secchitiello; IS, Ischia; AA, Acciaroli A; AB, Acciaroli B.

Sites were characterized by a high diversity of CA, ranging from a minimum of 6 CA taxa at Punta Campanella to a maximum of 12 at Capri, Ischia, and Acciaroli B (Figure 8). *Lithothamnion minervae* was the most abundant species at each site (23–38%), except at Acciaroli A (15%), where the most abundant was *L. corallioides* (69%).

## 4. Discussion

### 4.1. Characterization of Rhodolith Beds

A spatial variability of rhodolith covers was observed within the different sites along the ROV video-routes. Spatial heterogeneity, with high cover of rhodoliths interspersed with patches of sediments, has been already reported for deep Mediterranean RBs and seems to be mainly related to seafloor morphology and/or bottom currents [9,72,86].

RBs were found in Capri, Ischia, and Cilento with more than 10% of the mobile substratum covered by live rhodoliths [2]. Secchitiello and Punta Campanella cannot be considered as proper RBs, since they were mostly characterized by sparse rhodoliths (mean cover <10%). However, these sites showed higher local rhodolith covers (>10%) often associated with small accumulation patches and ripple marks, which indicate water motion over the sea floor [1,51].

The bathymetric range of the investigated RBs well compares with other Mediterranean beds, which mainly occur at 30–75 m of depth [1,4]. Rhodolith cover was lower at deeper sites (60–79 m), compared to the shallower ones (40–59 m), probably because of light irradiance reduction with depth. Higher rhodolith covers were also detected in concurrence with coarser sediments. This could be related to the presence of suitable sediments (e.g., gravel) for algal spore settlement and RBs development [1,2,7,67,87]. Pralines were the prevalent morphotype in almost all sites (except at Acciaroli A), suggesting the presence of high water energy [2,9,10,88,89].

We report the occurrence of a RB off the Capri Island, characterized by the highest rhodolith cover (> 65%) compared to the other sites, with a low percentage of dead thalli (16%) suggesting a good status of the bed. The high cover of rhodoliths might be explained by the abundance of coarse sediments as suitable substrate for CA settlement [7,10].

Acciaroli A was characterized by the prevalence of unattached branches, despite being mostly dead (76%) and fragmented. The presence of beds offshore the Cilento peninsula was already reported on submerged terraces between 42 to 52 m [72], mainly composed by a superficial layer of *Lithothamnion corallioides*, over a thick dead and sub-fossil rhodolith deposit. At Acciaroli B, the image analysis showed a lower cover of dead thalli (14%) compared to the live ones. Moreover, although unattached branches were observed, pralines were the most abundant morphotype in all the examined video frames. These two sites are most likely part of a unique and wide RB off the Cilento coast, as suggested by SSS data, structured by different rhodolith morphotypes and composed by a healthy ”live” part (i.e., the Acciaroli B site) and a “dead” (or fossil) part (i.e., the Acciaroli A site).

An extended area with rhodoliths off the Ischia Island has already been described by [69,70]. They described a 1-mile-long RB located off Ischia between 50 and 65 m depth, characterized by all the three rhodolith morphotypes, with a predominance of unattached branches of *Phymatolithon calcareum* and *Lithothamnion corallioides*. According to these previous studies, we also found in our investigation site all the three morphotypes, although the unattached branches cover was very low (<10%) and *P. calcareum* and *L. corallioides* were not predominant. Moreover, despite it is common for live RBs to be accompanied by a variable quantity of dead rhodoliths and their fragments [90], we observed a high percentage of dead thalli (26%). This finding was not reported in previous studies for Ischia [69,70], and we hypothesize that it could be related to the high fraction of fine sediments observed, which might have caused burial phenomena [4,67,91]. However, further investigations would be necessary to evaluate the entity and the possible progression of this algal mortality and its causes.

### 4.2. Rhodolith Morphology

The investigated RBs were similar with each other and with other deep RBs of the Mediterranean Sea [9,10,86,92], characterized by the pralines’ facies, as described by Pérès and Picard [93]. CA growth-forms ranged from encrusting/warty to lumpy/fruticose, with several protuberances and branches degrees, mainly depending on the CA species composing the rhodolith [10].

Surface currents within the Gulf of Naples have been deeply investigated [94,95,96,97,98,99], and there are several models describing their dynamics within the Campania coastal system [100,101,102]. However, macroscale hydrodynamic measurements at the sea-bottom lack for this area, and hydrodynamic models have limitations in simulating the near-bottom currents due to boundary conditions for numerical resolution. However, rhodoliths can represent, to some extent, a proxy of bottom currents [67]. The spheroidal to ellipsoidal shape of the rhodoliths analysed in this study and the lack of discoidal shapes seem to suggest an equilibrium between a regular frequency of overturning associated with moderate/high water energy, which assures the homogeneous growth of the thallus [8,10,67,87,103]. Moreover, the reduced sizes of the rhodoliths composing the Campania beds (i.e., from 21.8 mm ±8.5 in Capri to 10.6 mm ±6.1 in Secchitiello), according to literature [2,10,88,89], seem to support the presence of an elevated water energy in all sites.

### 4.3. Taxonomic Composition of Coralline Algae

This study provided a first picture of the CA diversity characterizing deep RBs off the Campania coast. The beds characterized by the highest number of CA were Capri, Ischia, and Acciaroli B, with 12 taxa each. The genus *Lithothamnion* was the most abundant, representing more than 50% of the total abundance of taxa in each site. *L. minervae* was the dominant species at each site (23–38%), except at Acciaroli A (15%) that was mostly composed by unattached branches of *L. corallioides* (69%). At all sites, except Punta Campanella and Secchitiello, we found both species, *P. calcareum* and *L. corallioides,* protected by the Habitats Directive (92/43/EEC). These results confirm the studies on Mediterranean RBs that report a high CA species diversity [9,53,70,71,72,86,104,105,106,107,108,109,110], highlighting the elevated scientific and ecological value of these remote biodiversity hot spots in the Mediterranean Sea.

## 5. Conclusions

This work provides new insights at large scale on deep Mediterranean RBs distribution and composition off the Campania coast (eastern Tyrrhenian Sea). The occurrence of a new RB offshore Capri Island, characterized by the highest rhodolith cover (>65%) compared to the other studied sites, is reported. The high rhodoliths’ cover might be related to the co-occurrence of coarse sediments and shallow depth. Small pralines (ca. 2 cm) dominated all the studied RBs, except at Acciaroli A, that was mostly composed by dead fragments of unattached branches. Ischia RB was also characterized by a conspicuous cover of dead rhodoliths. The collected rhodoliths were spheroidal to ellipsoidal in all sites, probably indicating a moderate/high hydrodynamism. Growth-forms varied from encrusting/warty to fruticose/lumpy, mainly related to the CA species composing the rhodoliths. CA identification highlighted a high taxonomic diversity (i.e., 13 taxa).

The investigated RBs are particularly valuable since the two species *Phymatolithon calcareum* and *Lithothamnion corallioides*, included and protected by the Habitats Directive (92/43/EEC), have been detected in all RBs. This study supports the ecological value of these highly biodiverse yet poorly explored deep macroalgal communities and represents a baseline for their future management and conservation.

## Figures and Tables

**Table 1:** Resuming data of sampling sites.

Site	Latitude	Longitude	Time Period	ROV Routes’ Depth	Grab Samples’ Depth
Capri (CA)	40°32’20.545’’ N	14°13’17.653’’ E	Jul-18	42–64 m	55–59 m
Punta Campanella (PC)	40°34’26.08’’ N	14°19’19.049’’ E	Jul-18	53–62 m	52–62 m
Secchitiello (SE)	40°34’45.538’’ N	14°17’15.261’’ E	Aug-18	70–78 m	68–72 m
Ischia (IS)	40°46’34.774’’ N	13°53’49.401’’ E	Apr-17	58–73 m	62–72 m
Acciaroli A (AA)	40°8’9.582’’ N	15°3’46.011’’ E	Jul-17	49–52 m	48–49 m
Acciaroli B (AB)	40°6’37.685’’ N	15°3’39.149’’ E	Jul-17	65–73 m	62–65 m

**Table 2:** Summary of prevalent (>75% of analysed video frames) depth interval and substrate type at the different sites. “shallow”, 40–59 m; “deep”, 60–79 m.

Site	Depth Interval	Substrate Type
Capri (CA)	Shallow	Coarse
Punta Campanella (PC)	Shallow	Coarse
Secchitiello (SE)	Deep	Fine
Ischia (IS)	Deep	Fine
Acciaroli A (AA)	Shallow	Coarse
Acciaroli B (AB)	Deep	Fine
